# Integrin-linked kinase expression in myeloid cells promotes colon tumorigenesis

**DOI:** 10.3389/fimmu.2023.1270194

**Published:** 2023-11-21

**Authors:** Afsar U. Ahmed, Saleh Almasabi, Ron Firestein, Bryan R.G. Williams

**Affiliations:** Centre for Cancer Research, Hudson Institute of Medical Research, Department of Molecular and Translational Science, Monash University, Clayton, VIC, Australia

**Keywords:** ILK, myeloid, CRC, CAC, Apc min/+, M2 polarization, CD8, Foxp3

## Abstract

Colorectal cancer (CRC) is one of the most common forms of cancer worldwide and treatment options for advanced CRC, which has a low 5-year survival rate, remain limited. Integrin-linked kinase (ILK), a multifunctional, scaffolding, pseudo-kinase regulating many integrin-mediated cellular processes, is highly expressed in many cancers. However, the role of ILK in cancer progression is yet to be fully understood. We have previously uncovered a pro-inflammatory role for myeloid-specific ILK in dextran sodium sulfate (DSS)-induced colitis. To establish a correlation between chronic intestinal inflammation and colorectal cancer (CRC), we investigated the role of myeloid-ILK in mouse models of CRC. When myeloid-ILK deficient mice along with the WT control mice were subjected to colitis-associated and APC^min/+^-driven CRC, tumour burden was reduced by myeloid-ILK deficiency in both models. The tumour-promoting phenotype of macrophages, M2 polarization, *in vitro* was impaired by the ILK deficiency and the number of M2-specific marker CD206-expressing tumour-associated macrophages (TAMs) *in vivo* were significantly diminished in myeloid-ILK deficient mice. Myeloid-ILK deficient mice showed enhanced tumour infiltration of CD8+ T cells and reduced tumour infiltration of FOXP3+ T cells in colitis-associated and APC^min/+^-driven CRC, respectively, with an overall elevated CD8+/FOXP3+ ratio suggesting an anti-tumour immune phenotypes. In patient CRC tissue microarrays we observed elevated ILK+ myeloid (ILK+ CD11b+) cells in tumour sections compared to adjacent normal tissues, suggesting a conserved role for myeloid-ILK in CRC development in both human and animal models. This study identifies myeloid-specific ILK expression as novel driver of CRC, which could be targeted as a potential therapeutic option for advanced disease.

## Introduction

Colorectal cancer (CRC) is among the most prevalent forms of cancer worldwide and the second-leading cause of cancer-related deaths in developed countries (1–4). Although colonoscopy screening is an effective way to detect and prevent CRC by surgical excision of precancerous adenomas (5), the 5-year survival rate is extremely low for patients with advanced disease accounting 70% of all detected cases (6). Despite being one of the most common cancers in Western populations, there remain few effective therapies for treating advanced CRC.

CRC originates from intestinal epithelial cells (IECs) due to the loss of tumour suppressor genes such as the adenomatous polyposis coli (*Apc*) gene (7). While hereditary CRC due to genetic alterations accounts for about 5% of cases (8), most risk factors are related to Western lifestyles causing >75% of CRC in patients with few or no genetic risk factors (8). Several studies implicate chronic inflammatory stimuli as a risk factor for developing CRC, particularly ulcerative colitis (UC), a form of inflammatory bowel disease (IBD), leading to so-called colitis-associated cancer (CAC) (9, 10). There are several animal models that have been developed to study colon cancer (11–14). Among them, CAC provides a ‘proof of concept’ model to better understand how chronic inflammation promote tumour initiation and progression (15) whereas the APC^min/+^ mouse carrying a heterozygous germ-line mutation at codon 850 of the *Apc* gene is one of the most commonly used models to study spontaneous (hereditary) CRC (16).

Tumour-associated immune cells comprise an essential component of the tumour microenvironment (TME) that develops from chronic inflammatory conditions that emerges from germ-line mutations (17). Both innate and adaptive immune cells participate in the immune landscape of the TME. These cells strongly influence tumorigenesis which can be either promoted or suppressed depending on the dominating cell type and polarization profile of the infiltrating immune cells (18). A large number of studies have demonstrated a key role for myeloid cells in CRC as well as other cancer types (18). Depending on their functional status, tumour-infiltrating macrophages can influence both positive and negative outcomes of CRC. Macrophages have been grouped into classically activated M1 and alternatively activated M2 macrophages (19). In the context of TME, M1 macrophages are considered to be tumoricidal whereas M2 macrophages show pro-tumoral phenotype (20). Even though their function seems to vary depending on the tissue, location and TME, tumour-associated macrophages (TAMs) support rather than hinder tumorigenesis. The molecular determinants of the pro-tumoral phenotypes of tumour myeloid cells could lead to novel therapeutic innovations in CRC. However, the detailed molecular mechanisms underlying tumour-promoting macrophage polarization are still poorly understood.

Integrin-linked kinase (ILK) is a multifunctional serine/threonine protein pseudokinase expressed ubiquitously in mammalian tissues and is involved in focal adhesion formation and function (21). ILK regulates many integrin-mediated cellular processes, including growth, proliferation, migration, invasion, and tissue homeostasis (22). Consequently, global genetic deletion of ILK is embryonic lethal due to cytoskeletal and cell attachment defects. Overexpression of ILK is also implicated in pathophysiological conditions, including cancer. ILK overexpression is a feature in the progression of many cancers such as colon, gastric, prostate, breast, melanoma as well as leukaemia (23–26). Moreover, intestinal epithelial cell (IEC) -specific ILK deletion in mice reduced both colitis and CAC, suggesting an integral role for IEC-ILK in intestinal inflammation and CRC (27, 28). Despite considerable evidence linking ILK to cancer, a mechanistic understanding of the role for ILK in cancer progression is yet to be fully realized. Based on our earlier study demonstrating ILK as a pro-inflammatory molecule (29), we investigated the role myeloid-specific ILK in a mouse model of colitis using myeloid-ILK deficient (M-ILK KO) mice., In this model the pathology of experimental colitis was significantly reduced by myeloid ILK deficiency with the pro-inflammatory signalling during intestinal inflammation dependent on myeloid-ILK (30). Given the correlation between chronic intestinal inflammation and CRC, in this study we investigated the effect of myeloid-ILK deficiency in mouse models of CAC and APC^min/+^-driven CRC. We show that myeloid-ILK deficiency decreases both AOM/DSS-induced and APC^min/+^-driven colon tumour burden. Our results indicate that ILK is required for macrophage M2 polarization *in vitro* and the expression of M2-specific marker CD206 in tumour-associated macrophages (TAMs) *in vivo*. Myeloid-ILK deficiency also enhances tumour-infiltration of CD8+ T cells and reduces FOXP3+ T cells in CAC and APC^min/+^-driven CRC, respectively, with a significantly elevated CD8+/FOXP3+ ratio indicating a tumoricidal impact in both models. Examination of human CRC tissue microarrays (TMAs), indicate that ILK+ myeloid cells (ILK+ CD11b+) are significantly elevated within the tumour compared to the adjacent normal tissues, suggesting a role of myeloid-ILK in human CRC development consistent with the results in the mouse models. Our study reveals an integrated role for myeloid-specific ILK expression in colon tumorigenesis and highlights the therapeutic potential of targeting ILK in CRC.

## Results

### ILK expression in myeloid cells promotes colitis-associated and spontaneous colon tumorigenesis

Our earlier studies demonstrated that the myeloid-specific ILK is required for intestinal inflammation during DSS-induced colitis (30). Since chronic colitis is a significant risk factor for development of CRC, we investigated whether myeloid-ILK deficiency ameliorates the colitis-associated cancer (CAC) model, also known as the AOM/DSS model (31). Azoxymethane (AOM) is procarcinogen which is commonly used to induce adenoma growth in the distal colon of rodents and AOM/DSS induces a model of carcinogen-induced intestinal tumorigenesis that reliably induces non-invasive colonic tumours in WT mice.

**Figure 1:**
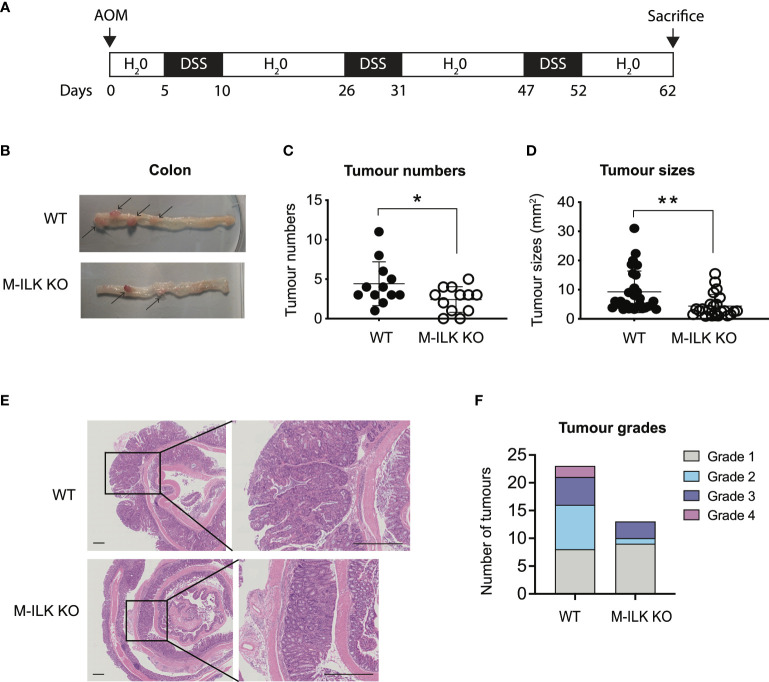
Loss of ILK in myeloid cells reduces colonic tumour formation in AOM/DSS model. **(A)** A schematic protocol of the AOM/DSS mouse model of CRC used in this study. **(B)** Representative images of the dissected colon in WT and myeloid-ILK deficient (M-ILK KO) mice following the AOM/DSS model. Arrows indicate polyps. Briefly, 12-week-old mice were subjected to AOM/DSS treatment which is based on a single i.p. injection of a carcinogen (AOM) (12.5 mg/kg) at day 0 followed by 3 cycles of inflammatory agent DSS (2%) treatment in the drinking for 5 days and a subsequent 10-days recovery period without DSS started from day 5 to day 62. All mice were sacrificed at day 62 for post-mortem examination. **(C)** Numbers of visible polyps in the colon intestine of mice with indicated genotypes are shown. Results are representative of at least three independent experiments and are shown as mean ± SD., *n* = 12 per group. **(D)** Colon tumour sizes of mice with the indicated genotypes are shown. Results are representative of at least three independent experiments and are shown as mean ± SD., *n* = 53 (WT tumours) and *n* = 29 (M-ILK KO tumours). **(E)** Representative of H&E-stained sections of colonic tissue with tumours from WT and M-ILK KO mice are shown (scale bars, 200 µm). **(F)** Blinded histological scoring of colon adenocarcinomas from WT and M-ILK KO mice, *n* = 23 (WT tumours) and *n* = 13 (M-ILK KO tumours). *, *p* < 0.05; **, *p* < 0.01, Student’s *t* test.

To investigate the role of myeloid-ILK in colitis-associated tumorigenesis, both wild-type (WT) or M-ILK KO mice were treated with AOM and DSS (**Figure 1A**). Briefly, mice were given a single i.p. injection of the mutagen AOM, after which they received drinking water containing 1% DSS in three rounds of 5-day periods that were interspersed with periods in which they received normal water. According to the post-mortem examination following the 62-day long AOM/DSS model, the number of visible intestinal polyps and their sizes in M-ILK KO -deficient mice was reduced significantly relative to control mice, suggesting that myeloid-specific ILK loss suppresses colitis-associated colon tumorigenesis (**Figures 1B-E**).

**Figure 2:**
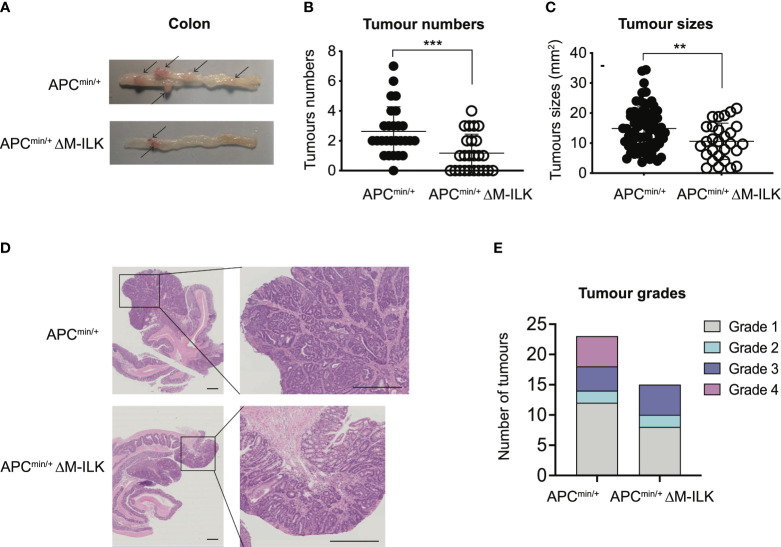
Myeloid-ILK deletion attenuates colonic tumorigenesis in APC^min/+^ model. **(A)** Representative images of the dissected colon in APC^min/+^ and APC^min/+^ΔM-ILK mice sacrificed at 16-weeks of age. Arrows indicate polyps. **(B)** Numbers of visible polyps in the colon intestine of mice with indicated genotypes are shown. Results are representative of at least three independent experiments and are shown as mean ± SD, *n* = 27 (APC^min/+^) and *n* = 23 (APC^min/+^ΔM-ILK). **(C)** Colon tumour sizes of mice with the indicated genotypes are shown. Results are representative of at least three independent experiments and are shown as mean ± SD, *n* = 71 (APC^min/+^) and *n* = 28 (APC^min/+^ΔM-ILK). **(D)** Representative of H&E-stained sections of colonic tissue with tumours from WT and M-ILK KO mice are shown (scale bars, 200 µm). **(E)** Blinded histological scoring of colon adenocarcinomas from WT and M-ILK KO mice, n = 23 (APC^min/+^) and n = 15 (APC^min/+^ΔM-ILK). **, *p* < 0.01; ***, *p* < 0.001, Student’s *t* test.

While tumours of all genotypes were predominantly low-grade adenocarcinomas, the proportion of tumours that progressed to high-grade adenocarcinomas was relatively higher in WT mice compared to their myeloid-ILK deficient counterparts (**Figure 1F**), suggesting a role for myeloid-ILK in tumour progression. Based on these observations, we investigated further whether myeloid-ILK plays a role in colitis- independent colon tumour development. Consequently, we used a genetic model of spontaneous adenomatous polyposis driven by APC^min/+^. To test the effect of myeloid-ILK deficiency in APC^min/+^ model, we generated myeloid-ILK deficient APC^min/+^ mice (ILKfl/fl;LysMCre; APC^min/+^, referred as APC^min/+^ΔM-ILK hereafter). Analysis of 4-month-old APC^min/+^ΔM-ILK along with the WT APC^min/+^ control mice, showed both tumour incidence and size were significantly decreased in APC^min/+^ΔM-ILK compared to the WT control (**Figures 2A–D**). Similar to the AOM/DSS model, the proportion of APC^min/+^ -driven tumours that progressed to high-grade adenocarcinomas was reduced in myeloid-ILK deficient APC^min/+^ mice compared to the WT control (**Figure 2E**). Collectively, these results demonstrate marked tumour suppression by myeloid-ILK deficiency in both carcinogen/inflammation and oncogene-driven models of colon tumorigenesis.

### Myeloid-ILK deficiency impedes tumour-driven body weight loss, poor survival and splenomegaly in APC^min/+^ mice

**Figure 3:**
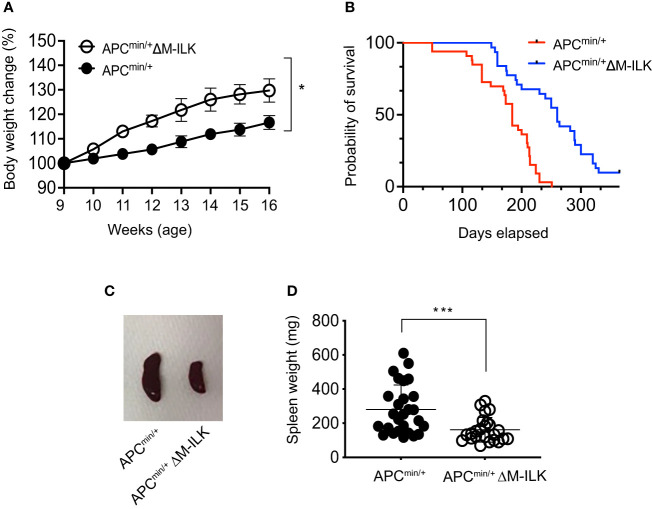
Myeloid-ILK deficiency reverses body weight loss, improves survival and reduces splenomegaly in *Apc^min/+^* mice. **(A)** Changes in body weight in both APC^min/+^ and APC^min/+^ΔM-ILK mice are expressed as percentage of body weight at 9 weeks of age. Body weights of randomly selected mice from each genotype were measured weekly from week 9 to week 17, n = 10 per group. Results are representative of at least three independent experiments and are shown as mean ± SD. **(B)** Both APC^min/+^ and APC^min/+^ΔM-ILK mice were followed for long-term survival. Survival probability was analyzed using the Kaplan-Meier estimate, and differences were evaluated using the log-rank test, *n* = 33 (APC^min/+^) and *n* = 31 (APC^min/+^ΔM-ILK). **(C)** Representative images of spleen from APC^min/+^ and APC^min/+^ΔM-ILK mice sacrificed at 16-weeks of age. **(D)** Spleen weights (mg) are shown for both APC^min/+^ and APC^min/+^ΔM-ILK mice sacrificed at 16-weeks of age, *n* = 27 (APC^min/+^) and *n* = 24 (APC^min/+^ΔM-ILK). Results are representative of at least three independent experiments and are shown as mean ± SD. *, *p* < 0.05; ***, *p* < 0.001, Student’s *t* test.

The APC^min/+^ mouse is not only an established model of CRC but also presents with cancer cachexia, a wasting syndrome due to body weight loss associated with the skeletal muscle wasting with or without the loss in adipose tissue (32–34). An advantage of this mouse model is the gradual progression of tumour development and muscle wasting that is more physiologically related to human disease. The genetic predisposition in APC^min/+^ mice becomes apparent as early as 3 weeks of age and most of the adenomas start to appear in APC^min/+^ mice at 5–8 weeks of age (35). In order to monitor the effect of myeloid-ILK deficiency on the development of genetic predisposition in APC^min/+^ mice, body weight changes in both APC^min/+^ and APC^min/+^ΔM-ILK mice were measured from 5 to 16 weeks of age. As shown in **Figure 3A**, APC^min/+^ΔM-ILK mice started to show an elevated body weight compared to APC^min/+^ mice from the age of 9 weeks onwards. The body weight of APC^min/+^ΔM-ILK mice was increased by approximately 10% from 11 to 16 weeks of age compared to the same aged-APC^min/+^ mice.

Since myeloid-ILK deficiency improves body weight loss in APC^min/+^ mice (**Figure 3A**) and cancer-associated cachexia is associated with a poor outcomes with a reduced survival rate (33, 36), it was of interest to determine whether myeloid-ILK deficiency confers a protective effect and prolonged survival in APC^min/+^ mice. As shown in **Figure 3B**, the mortality rate of APC^min/+^ΔM-ILK mice was significantly reduced relative to APC^min/+^ control mice. APC^min/+^ΔM-ILK mice had a median survival of 253.7 days compared to 174.5 days for APC^min/+^ mice. It was previously reported that the spleens were enlarged (known as splenomegaly) in tumour-laden APC^min/+^ mice due to vigorous splenic haematopoiesis (37, 38). In fact, the splenomegaly process was positive correlated with the tumour development in the APC^min/+^ mice (39). Similarly, we observed splenomegaly in APC^min/+^ mice following post mortem examination at 16 weeks of age. The spleen size and weight between APC^min/+^ and APC^min/+^ΔM-ILK mice, (sizes shown as representative images in **Figure 3C**) were significantly reduced in APC^min/+^ΔM-ILK mice compared to the ILK-sufficient APC^min/+^ control mice (**Figure 3D**). Overall, these results demonstrate that myeloid-ILK pays critical roles in APC^min/+^-driven physiological abnormalities including body weight loss, splenomegaly and poor survival.

### ILK promotes M2 macrophage polarization

**Figure 4:**
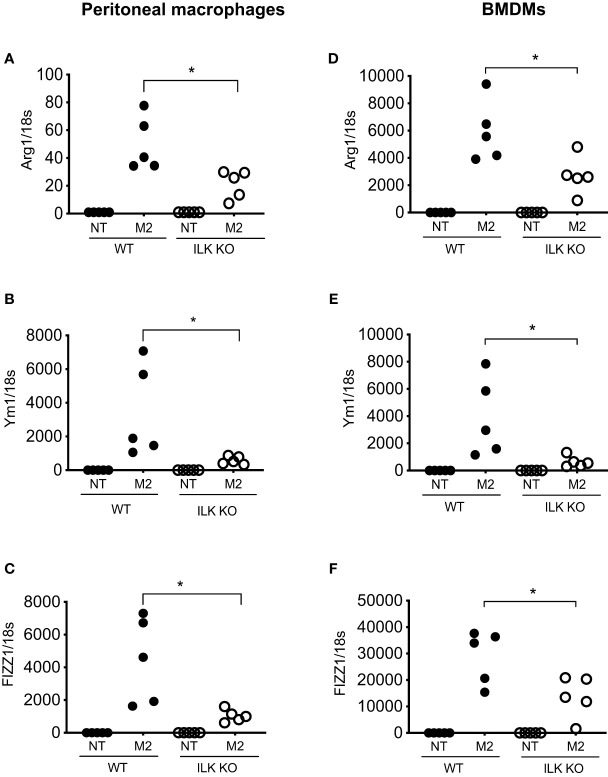
ILK promotes M2 macrophage polarization. q-RT-PCR analyses of M2-specific genes *Arg1*, *Ym1* and *FIZZ1* in both peritoneal **(A–C)** and bone-marrow derived macrophages (BMDM) **(D–F)** isolated from WT and myeloid-ILK deficient (M-ILK KO) mice are shown. Analyses were carried out under both resting and M2-polarised conditions (refer to Materials and Method section). Results are representative of three independent experiments and are shown as mean ± SD. *n* = 5 per genotype. *, *p* < 0.05; Student’s *t* test.

The impact of myeloid-ILK deficiency on colon tumour development suggests a macrophage-intrinsic role of ILK affecting tumour growth via the regulation of macrophage phenotypes. Macrophages exhibit a wide phenotypic diversity and exert multiple pathobiological effects on tumour growth. The ability of macrophages to adapt to their environment has led to the identification of two main polarized phenotypes – classically activated macrophages (M1) and alternatively activated macrophages (M2) (19). M1 macrophages are characterized by the expression of many pro-inflammatory cytokines and a high tumoricidal capacity, while alternatively activated M2 macrophages perform immunosuppressive functions and tumour promotion. The distinction between M1 and M2 cells has been verified using gene profiles, which support this phenotypic and functional change (40). The classical M1 activation is driven by the stimulation of TLR ligands (e.g. LPS) and IFN-γ (41, 42). Classically activated macrophages upregulate the expression of proinflammatory cytokines (e.g. TNF-α) and mediators such as iNOS and CCL2. Alternative/M2 polarization is induced by exposure to IL-4 and IL-13 cytokines that bind to a common receptor subunit, IL-4R (43). M2 macrophages up-regulate expression of the mannose receptor (CD206) as well as macrophage-specific genes including *Arg1*, *Ym1* and *Fizz1* (44). To examine whether ILK plays any role in macrophage polarization, both peritoneal and bone-marrow derived macrophages (BMDMs) from WT and M-ILK KO mice were isolated and polarized *in vitro* to the M1 phenotype by exposure to LPS and IFN-γ as well as the M2 phenotype by exposure to IL-4 and IL-13 cytokines. As per our observation, during M1 polarization, only TNF-α expression was affected by ILK deletion in both BMDMs and peritoneal macrophages which correlates with our earlier observation (29) but the expression levels of both iNOS and CCL2 remained unaffected, indicating that macrophage M1 polarization is only partially impacted by ILK deletion (**Supplementary Figure 1**). Western blot analysis of iNOS protein levels in both WT and ILK-KO BMDMs during M1 polarization further confirms that ILK is dispensable for iNOS expression (**Supplementary Figure 2**). Next, we examined whether the tumour-promoting M2 phenotypes are affected by deletion of ILK in macrophages. Accordingly, Analyses of M2-specific gene expression markers (*Arg1*, *Ym1* and *Fizz1*) showed a consistent impairment in ILK KO cells compared to the WT cells (**Figure 4**), suggesting impeded M2 polarization in ILK KO cells. Reduced expression levels of *Arg1* observed in ILK-KO cells also correlated with reduced levels of Arginase-1 protein in ILK-KO BMDMs during M2 polarization (**Supplementary Figure 3**). Our results also correspond with our earlier report indicating a correlation between ILK expression and markers of M2 macrophages (45). These observations establish that ILK is required for macrophage M2 polarization.

### Myeloid-ILK deficiency is associated with reduced infiltration of CD206+ cells in tumours

**Figure 5:**
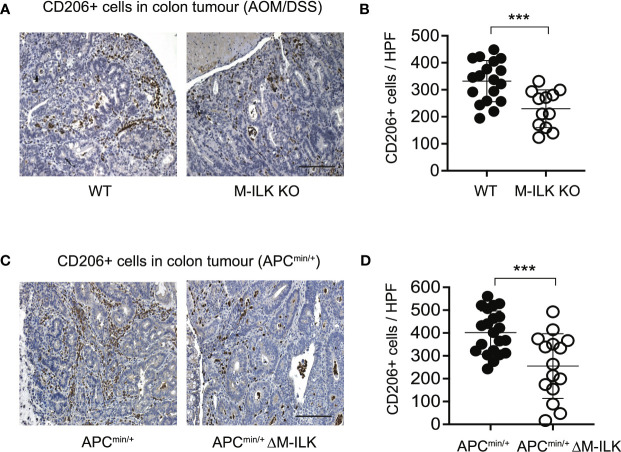
Myeloid-ILK deficiency is associated with reduced infiltration of CD206+ cells in tumours. **(A)** Representative immunohistochemistry images of colon tumours showing CD206+ cells of indicated mice subjected to the AOM/DSS model and **(B)** quantification of CD206+ cells in high-powered fields (HPFs, original magnification x400) across 5 mice per genotype, *n* = 18 (WT) and *n* = 18 (M-ILK KO) HPFs. **(C)** Representative immunohistochemistry images of colon tumours showing CD206+ cells of APC^min/+^ and APC^min/+^ΔM-ILK mice, and **(D)** quantification of CD206+ cells in high-powered fields (HPFs) across 5 mice per genotype, *n* = 21 (APC^min/+^) and *n* = 15 (APC^min/+^ΔM-ILK) HPFs. Results are shown as mean ± SD. Scale bars, 100 µm; ***, *p* < 0.001, Student’s *t* test.

Cancer progression is a complex multistep process which involves interactions of neoplastic cells with various immune and non-immune cells (46). TAMs that infiltrate into TME play a complex role in tumour progression through their interactions with neoplastic cells (47, 48). TAMs are therefore characterized as M2 polarized macrophages in response to cues from tumour cells and activated lymphocytes. The mannose receptor (CD206), a proteolytic and widely used marker for alternatively activated type 2 (M2) macrophages (44), is highly expressed on TAMs. Since ILK deficiency impairs macrophage M2 polarization *in vitro* (**Figure 4**), we wanted to investigate further whether myeloid-ILK deficiency impairs TAMs M2 polarization *in vivo*. To do this, we quantified the tumour-infiltrated CD206+ cells via IHC using tumour sections. As shown in **Figure 5**, the tumour infiltration of CD206+ cells was significantly reduced in both AOM/DSS and APC^min/+^-driven models of CRC, suggesting that ILK is required for the tumour promoting role of TAMs.

### Myeloid-ILK deficient mice demonstrate enhanced tumour-infiltration of CD8+ T cells in AOM/DSS and reduced tumour-infiltration of FOXP3+ T cells in APC^min/+^ models

**Figure 6:**
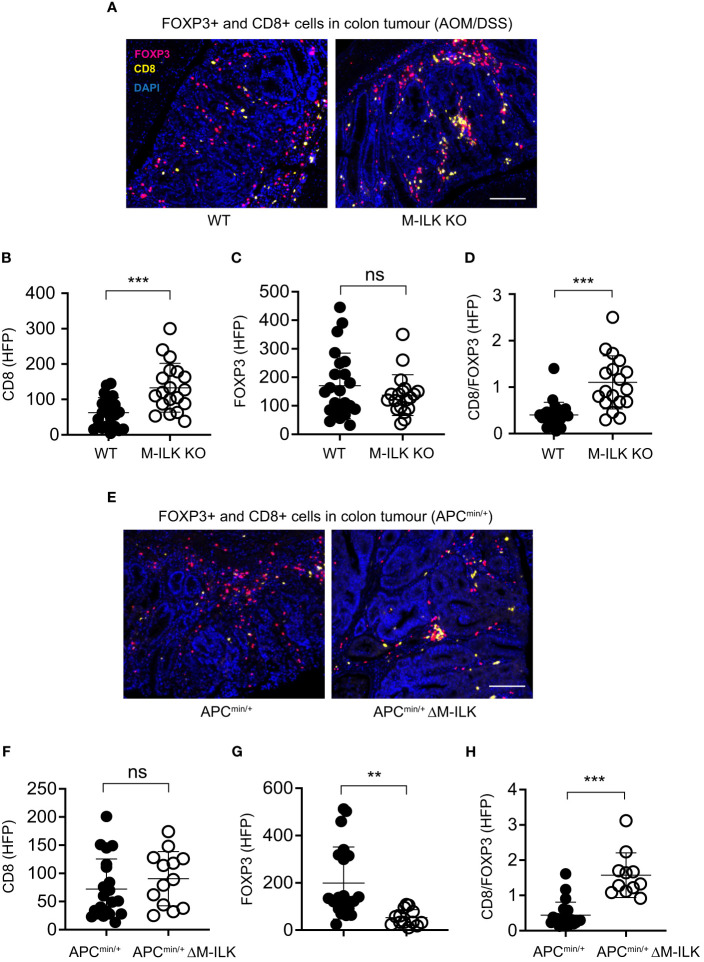
Myeloid-ILK deficiency enhances tumour-infiltration of CD8+ T in AOM/DSS model and reduces tumour-infiltration of FOXP3+ T cells in APC^min/+^ model. **(A)** Representative immunofluorescence images of colon tumours showing infiltration of FOXP3+ and CD8+ T cells in tumours of indicated mice subjected to the AOM/DSS model. Pink, FOXP3+ cells; yellow, CD8+ cells; and blue, DAPI. Quantification of CD8+ T cells **(B)**, FOXP3+ T cells **(C)**, and the ratio of CD8+/FOXP3+ cells **(D)** in the AOM/DSS-driven colon tumours using the high-powered fields (HPFs, original magnification x400) across 4 mice per genotype, *n* = 23 (WT) and *n* = 19 (M-ILK KO) HPFs. **(E)** Representative immunofluorescence images of colon tumours showing infiltration of FOXP3+ and CD8+ T cells in tumours of APC^min/+^ and APC^min/+^ΔM-ILK mice. Pink, FOXP3+ cells; yellow, CD8+ cells; and blue, DAPI. **(B)** Quantification of CD8+ T cells **(F)**, FOXP3+ T cells **(G)**, and the ratio of CD8+/FOXP3+ cells **(H)** in APC^min/+^-driven colon tumours using high-powered fields (HPFs) across 4 mice per genotype, *n* = 21 (APC^min/+^) and *n* = 13 (APC^min/+^ΔM-ILK) HPFs. Results are shown as mean ± SD. Scale bars, 100 µm; **, *p* < 0.01; ***, *p* < 0.001, ns, not significant, Student’s *t* test.

Several studies have suggested integrally connected roles of myeloid cells with other immune cells including adaptive immune cells. For examples, based on 3D visualization of the CRC immune landscape, macrophages were found to be associated with the T cell network within tumours (49). Myeloid-derived suppressor cells (MDSCs) were shown to promote tumour growth by suppressing CD8+ T cell cytotoxic activity in the AOM/DSS model of CRC (15). Antibody-mediated depletion of macrophages decreased tumour-infiltrating both T cells and NK cells, thereby affecting anti-tumour immunity in a mouse model of cancer immunotherapy (50). Accordingly, to examine the effect of myeloid-ILK deficiency on the tumour-infiltration of T cells, the tumour-infiltrated regulatory FOXP3+ and cytotoxic CD8+ T cells in both models of CRC were quantified. As shown in **Figure 6** (A–C), myeloid-ILK deficiency resulted in enhanced filtration of CD8+ T cells without any impact of the infiltration of FOXP3+ T cells in the AOM/DSS model. In contrast, for the APC^min/+^ model, the tumour infiltration of FOXP3+ T cells were reduced by myeloid-ILK deficiency whereas the infiltration of CD8+ T cells remained unaffected (**Figures 6E–G**). In both models, the ratio of CD8+/FOXP3+ T cells in tumours were significantly elevated in myeloid-ILK deficient mice compared the ILK-sufficient controls (**Figures 6D, H**). Overall, these results indicate a role of myeloid-ILK in tumour promotion through regulation of the tumour-infiltration of T cells either by restraining tumour-suppressing CD8+ T cells or by enhancing tumour-promoting FOXP3+ T cells.

### CRC tumours from patients exhibit high ILK expression in both epithelial cells and infiltrating CD11b^+^ myeloid cells

**Figure 7:**
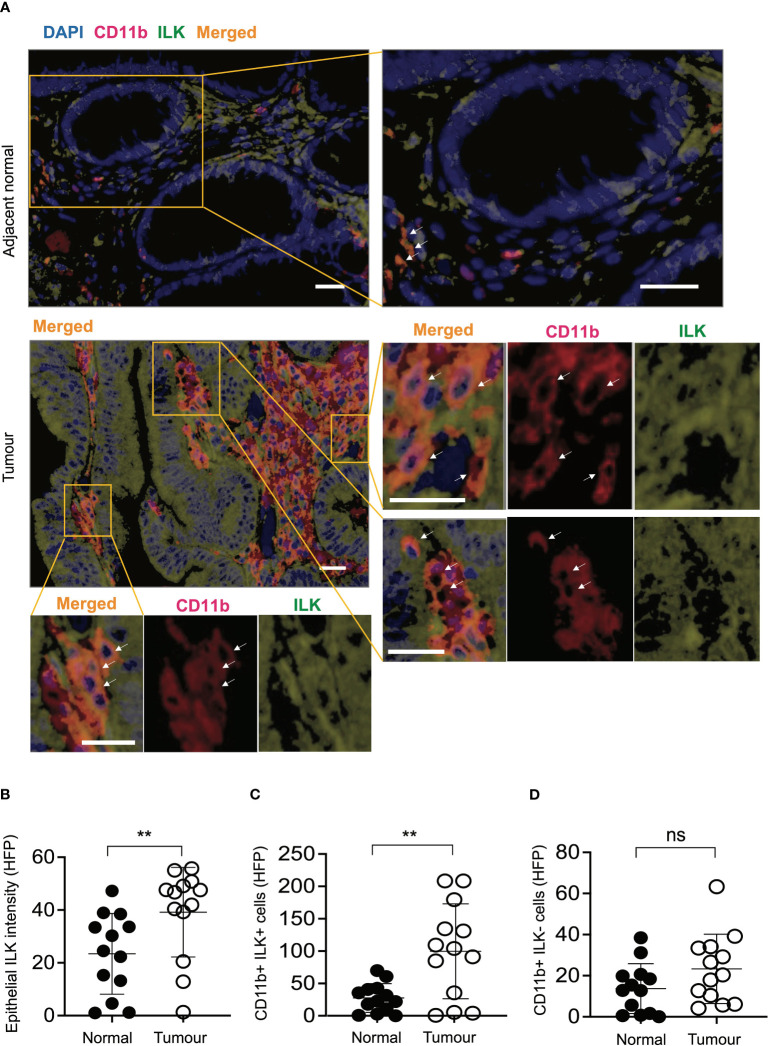
ILK expression is elevated in human CRC epithelial cells and infiltrating CD11b+ cells compared to the adjacent normal tissues. **(A)** CRC tissue microarray from patients (*n*=13) consisting of both tumours and their adjacent normal tissues were stained with immunofluorescence to investigate ILK expression (green) and CD11b marker for myeloid cell infiltration (red) in addition to the DAPI nuclear stain (blue). Orange colour in the merged image is an expression of both red CD11b and green ILK. The top panel is a representative microscopic image of adjacent normal tissue and the bottom panel is the tumour tissue showing ILK expression and infiltrating CD11b^+^ myeloid cells. Single-coloured images within the tumour section corresponding to CD11b+ cells (red) and ILK+ cells (green) show that merged signals (orange) are originated from the combined signals of both CD11b and ILK. Arrows indicate myeloid cells in both CD11b+ and merged images. The scale bar is 20μm. **(B-D)** Quantifications of epithelial ILK intensity **(B)**, CD11b+ ILK+ cells **(C)** and CD11b+ ILK- cells **(D)** in the tumours and their adjacent normal tissues. Results are shown as mean ± SD. Scale bars, 20 µm; **, *p* < 0.01, ns, not significant, Student’s *t* test.

In order to determine whether the observations in the above mouse studies correlate with human CRC, a CRC tissue microarray consisting of tumours and corresponding adjacent normal tissues of individual CRC patients (*n*=13) was examined. Based on immunofluorescence analyses of ILK+ and CD11b+ (myeloid) cells in both tumours and adjacent normal tissues, elevated expression of ILK was apparent not only in tumour epithelium but also in the tumour-infiltrating myeloid (CD11b+) cells (**Figure 7**). While the elevated ILK expression in tumour epithelium compared to the adjacent normal tissue is supported by published reports (27), we observed higher tumour-infiltration of ILK-positive myeloid (CD11b+ ILK+) cells as indicated by merged images from both CD11b+ and ILK+ cells (**Figure 7A**). In accordance with published literature highlighting a critical involvement of myeloid cells (51), we observed an elevated infiltration of myeloid cells in tumours (data not shown). Among the tumour-infiltrating myeloid (CD11b+) cells, our results indicate that the major proportion of these infiltrated CD11b+ cells are ILK+ compared to the ILK-deficient myeloid cells which are comparable to those in adjacent normal tissues (**Figures 7C, D**), suggesting myeloid-ILK as a prognostic marker for CRC in humans. Overall, these observations show that ILK is an integral player in the tumour-promoting role of myeloid cells.

## Discussion

Over the past several decades our understanding has been rapidly expanded in terms of epidemiology, aetiology, molecular biology and clinical aspects of CRC. Nevertheless, the incidence of CRC remains elevated with an annual 1.8 million new cases being diagnosed worldwide (52). Furthermore, CRC is no longer considered a disease primarily of Western populations only since rapid rises in incidence are also occurring in developing countries undergoing changes in diet and lifestyle (52). Colonoscopy screening is a gold standard method for detecting early stage CRC and nutrient-based chemoprevention such as calcium supplementation, vitamin D and folate could also curb CRC risk safely (5, 53, 54). While low dose aspirin has also been associated with a reduced risk of colorectal cancer in chemopreventative studies (55), there is a need for novel and efficacious drugs for advanced CRC. However, the existing medical treatments and preventive options are both currently insufficient to control the escalating rise in CRC (52). Therefore, it is critically important to fully understand the molecular drivers of CRC for the development of treatment and prevention strategies to counter the rise. In this study, we have identified myeloid-specific ILK as a critical driver of CRC which could potentially be developed as a as a novel therapeutic. However, the development of novel therapeutic options for CRC requires a deeper understanding of the mechanisms underlying tumour progression. Accumulating evidence suggests that tumour progression is largely governed by the complex interactions between tumours and their microenvironment. The involvement of immune cells in CRC progression and recurrence has been extensively demonstrated and the immune infiltrate composition has been characterised as the most heterogeneous and dynamic feature of CRC as its composition changes across tumour stages. Both adaptive and innate immune cells showed differential infiltration patterns along with tumour progression, implying the prognostic features of infiltrated immune cells (49). Further studies have defined a scoring system, termed Immunoscore (IS), based on the quantification of infiltrated cytotoxic and memory T cells. Interestingly, IS strongly predicts both tumour recurrence and patient survival (56). Moreover, higher IS was also associated with fewer metastases and, for the least-infiltrated metastasis, IS was also predictive of patient survival (57). Our study demonstrates the ratios of CD8/FOXP3 correlate with the tumour burdens in both AOM/DSS and APC^min/+^ models, indicating an impact of myeloid-ILK deficiency on the differential tumour-infiltration of T cells. The increased numbers of CD8 T cells showed a strong correlation with the reduced tumour burdens in both AOM/DSS- and APCmin/+ -driven mouse models, indicating an active status of CD8 T cells with an anti-cancer property. Further verification of active CD8 T cells status comprises the assessment of more than just one marker such as CD69, HLA-DR, IFN-γ, IL-2 and IL-12 (58, 59) and is unlikely to add anything new to our conclusions. Since the multiplex immunostaining will be challenging with additional markers along with existing CD8 and DAPI, it will require a completely different approach of extracting and flow cytometry analysis of tumour-infiltrated T cells and will be a significantly time-consuming exercise considering these mouse models. Overall, our observation also suggests a potential of role for myeloid-ILK in modulating the IS.

ILK is a focal adhesion protein which is ubiquitously expressed in both immune and non-immune cells. As an adaptor protein, it links the extracellular matrix with downstream signalling pathways and thereby it is implicated in many cellular processes such as survival, differentiation, proliferation, and gene expression. While under homeostasis ILK activation is transient as a biological switch for many signal transduction pathways, in pathological conditions (e.g. cancers) ILK is constitutively activated. Overexpression or constitutive activation of ILK is required for cell survival through the phosphorylation of Akt on Ser473, which also involve the activation of nuclear factor B (NF-κB) pathway (23, 24). Despite its central role in cell-extracellular matrix interactions, the mechanistic details of ILK upregulation is not fully understood yet but is considered to be cell type- and context-dependent (60, 61). For examples, our earlier report demonstrates a correlation between ILK overexpression and markers of M2 macrophages (45), indicating a cancer-promoting cue driving ILK upregulation. Even the mechanistic details could also vary within the same cell type depending on the stimulation. For example, we demonstrated earlier that ILK-driven phosphorylation of p65 on Ser536 and TNF-α synthesis are independent of PI3K/Akt pathway under LPS stimulation but they are dependent on the PI3K/Akt pathway during *H. pylori* infection (29). While the mechanistic details of ILK expression in myeloid cells are yet to be revealed, our study demonstrates a tumour-promoting role of myeloid-specific ILK expression.

CRC development is a complex, multistep process involving an intricate array of gene alterations. While it is not possible to have an universal mouse model to address this heterogenous disease, a number CRC mouse models were established which differ in their underlying mechanisms of tumorigenesis (62, 63). For example, AOM/DSS model induces several pathogenic pathways such as *Kras*, c-Myc and Apc/β-catenin. On the other hand, *Apc* mutation results in the activation of a complex together with *Apc*, Axin and GS3K, which leads to WNT/β-catenin signalling pathway. Regardless of the differences in the mechanisms of tumorigenesis, the hallmarks of immune cell infiltration within tumours seem to be mostly conserved given their impact on the TME and correlate with the tumorigenesis process (49). Accordingly, the overall CD8/FOXP3 ratio that we measured in the study correlates well with the tumour burden in both models. However, the differences in the number of infiltrated CD8+ and FOXP3+ cells that we observed between these models is most likely related to the underlying mechanism of tumorigenesis associated with these models. With respect to the details of tumour-associated immune signalling, there is not enough studies dedicated to comparison between these models. Nevertheless, the critical involvement of immune signalling in CRC development and progression is highlighted by recent literature showing a pivotal role of gut microbiota in both AOM/DSS and APCmin/+ models (63, 64). Even though the microbiota could vary from human to mice as well as within mice under various housing conditions, the outcomes of these models hold some relevance to the pathology of human CRC due to the randomness of carcinogen-induced mutations as well as the *Apc* mutation observed in over 70% of human CRC.

Among tumour-infiltrating immune cells, myeloid cells have been reported to be the most significant and abundant cell type. Several recent studies suggest a significant involvement of myeloid cells in tumour progression. For examples, the massive infiltration of granulocytic MDSCs (G-MDSCs) from the circulatory system to colonic mucosa is essential for the development of CAC tumorigenesis since the inhibition of G-MDSCs infiltration via CXCR2 deletion attenuated colitis-associated tumorigenesis (15). The levels of granulocytic myeloid cells were found to be elevated in both circulation and tumours of CRC patients (65). Myeloid-specific deletion of EGFR in mice resulted in fewer and smaller tumours in both APC^min/+^ and AOM/DSS models, and EGFR expression in myeloid cells but not in intestinal epithelial cells was associated with tumour metastasis and shorter patient survival time (66). The loss of IKKα kinase activity protected mice from intestinal tumour development via the suppression of M1-like myeloid cell infiltration rather than a direct impact on tumour cell proliferation (67). Increased oxidative stress in myeloid cells also promotes AOM-initiated colon tumorigenesis (68). Myeloid-specific p38α controls the tumour-promoting inflammatory microenvironment and promotes CAC (69). In a colorectal cancer liver metastasis model, the premetastatic niche in the liver was formed by MDSCs driven by the S1PR1–STAT3 signalling pathway in cancer cells (70). ScRNA-seq analyses on immune and stromal populations from CRC patients and mouse tumour models highlight conserved myeloid subsets as potential therapeutic targets (71). Transcriptomic analysis of tumour-infiltrating MDSCs also identified gene signatures in MDSC of poor prognosis (72). Tumor-resident *Aspergillus sydowii* promotes lung adenocarcinoma by inducing MDSCs expansion and activation (73), indicating a role of myeloid cells in intratumour microbiome-mediated lung cancer progression. Based on these diverse roles of myeloid cells integrated with cancer progression, the future prospect of myeloid cells as a potential therapeutic option has been widely recognised (51, 74). Our study further emphasises the critical involvement of myeloid cell biology in cancer progression and we demonstrate that ILK is required for macrophage M2 polarization. Macrophage polarization is a result of temporal changes in diverse signalling (NF-κB, IRF3, IRF5, STAT1/3/6, MEK and PPARχ/δ) and metabolic pathways (glucose, glycosaminoglycan and retinoic acid signalling) (41, 42, 75). Particularly, a shift in cellular metabolism including mitochondrial energy metabolism is more closely linked to macrophage functional changes. Future investigations on a diverse signalling pathways along with quantitative proteomics will be required to fully understand the mechanism of ILK-driven M2 polarization.

Over the last decade, immune cell therapeutic approaches to cancer have been dominated by both adoptive transfer of T cells and CAR T cell therapy, which have shown clinical efficacy only in the treatment of blood malignancies while the majority of solid tumours remains largely unresponsive. The lack of trafficking to the tumour site and the immunosuppressive nature of the TME have been key limitations to T cell-based therapies. Unlike T cells, the adoptive transfer of myeloid cells can overcome some of these limitations by ensuing more efficient trafficking to the TME. In relation to the immunosuppressive nature of TME, certain myeloid cell subsets (such as TAMs and MDSCs) are critical determinants of resistant to cancer therapies (76). As a result, targeting myeloid cell subsets as a potential therapeutic option for cancer has recently been the subject of intense research. However, developmental heterogeneity and functional redundancy of myeloid cells along with the lack of well-defined target specificity have made this approach challenging (51). In this study, we have identified the myeloid-specific ILK expression as a potential target for solid tumours such as CRC. Since integrins are the main cellular adhesion receptors that are implicated in nearly every step of cancer progression such as cancer cell migration and invasion, anchorage-independent survival and colonization of metastatic sites, the targeting of the integrin signalling has emerged as a therapeutic strategy in cancer (77). While the therapeutic potential of blocking integrin signalling has primarily been focused on cancer cells, its implication in the tumour-promoting roles of immune cells is yet to be fully explored. By identifying the myeloid-specific ILK as a potential driver of CRC, our study reveals a mechanism through which the tumour-promoting roles of myeloid cells could be exploited for clinical applications.

## Materials and methods

### Mice

ILK^fl/fl^;LysMCre (referred as myeloid-ILK deficient or M-ILK KO) mice were generated as described previously (30). ILK^fl/fl^;LysMCre;APC^min/+^ (referred as myeloid-ILK deficient APC^min/+^ or APC^min/+^ΔM-ILK) mice were generated by inter-breeding mice carrying ILK^fl/fl^;LysMCre and APC^min/+^. Aged-matched ILK-sufficient LysMCre and LysMCre;APC^min/+^ mice were used as wild-type (WT) controls for M-ILK KO and APC^min/+^ΔM-ILK mice, respectively. Genotypes of mouse litters were confirmed via PCR analysis using the tail DNA as described previously (30). The PCR analyses for ILK and LysMCre were carried out as described previously (30). Genotyping for APC^min/+^ was carried out using the following primers: APC-mutant (APC^min^) primer: 5′-TTCTGAGAAAGACAGAAGTTA-3′ and APC-common primer: 5′-TTCCACTTTGGCATAAGGC-3′, detecting the mutant APC allele (313 bp) which is only present in heterozygous APC mice. The PCR protocol for APC mice consists of an initial denaturing step of 5 min at 94 °C, followed by 30 cycles of 94 °C for 35 sec, 60 °C for 30 sec and 72 °C for 40 sec, and then a final extension at 72 °C for 5 min. All mice were generated and maintained in the same facility under pathogen-free conditions with a 12 hr light/dark cycle at the Monash Medical Centre Animal Facility, Melbourne, Australia (30). All animal breeding and experiment protocols were approved by the Monash University Animal Ethics Committee and adhered to the national guidelines provided by the Bureau of Animal Welfare, Victoria, Australia (30).

### Macrophage isolation and culture in conditioned media

BMDMs were isolated and cultured as described previously (30). Briefly, primary bone marrow cells were flushed from femurs of 8-weeks old mice and were differentiated to macrophages in RPMI 1640 supplemented with 20% L929–conditioned media, 10% FBS Glutamax and Pen/Strep over 7 d. Half of the culture media refreshed on day 5 and adherent macrophages were harvested by scraping on day 7. Peritoneal macrophages were harvested in PBS from unchallenged 8-weeks old mice, cultured in RPMI 1640 supplemented with 10% FBS Glutamax and Pen/Strep and adherent cells were used for experiments. The purity of the isolated macrophages was confirmed via the estimation of FITC-positive cells using FACS analysis. For M2 polarization, macrophages were stimulated with recombinant mouse IL-4 (50 ng/mL, Peprotech) and IL-13 (50 ng/mL, Peprotech) in RPMI 1640 supplemented with 10% FBS Glutamax and Pen/Strep. For M1 polarization, macrophages were stimulated with lipopolysaccharide (LPS) (100 ng/ml, Sigma) and recombinant mouse IFN-γ (50 ng/mL, Peprotech) in RPMI 1640 supplemented with 10% FBS Glutamax and Pen/Strep. After 24 hours, wells were washed with ice-cold PBS and adherent macrophages were detached from plates using a cell-scraper.

### Mouse models of colon cancer

The AOM/DSS model was performed as described by Tanaka et al. (31), with slight modifications. Briefly, 8-weeks old mice were given a single i.p. injection of the mutagen AOM (Sigma-Aldrich) at 12.5 mg/kg at day 0, after which at day 5 they received drinking water containing 1% DSS (30- 50 kDa, colitis grade, MP Biomedicals) over three cycles of 5-day periods that were interspersed with 16-day periods in which they received normal water. The entire model takes 62 days and all mice were sacrificed at day 62 for post-mortem examination. After the administration of DSS, all experimental mice were monitored daily for both general well-being and symptoms including weight loss, abnormal stool consistency, blood in stool and rectal prolapse. To minimize the degree of pain for experimental mice, the mouse intervention criteria were followed as per the guidelines of the ethics committee. For the APC^min/+^-driven colon cancer model, age- and gender-matched experimental APC^min/+^ΔM-ILK mice along with their WT counterparts were monitored once a week for both general well-being and symptoms related to tumour burden such as chronic weight loss, pale toes/feet and rectal prolapse. In case of any of these symptoms, mice were monitored daily and subjected to same intervention criteria as per the guidelines of the ethics committee. all experimental APC^min/+^ mice were sacrificed at 18 weeks of age for the post- mortem analysis of tumours. For the survival study, experimental APC^min/+^ mice were kept alive for up to 12 months as long as they remained healthy. At the end of both models, the colons were extracted, flushed with ice-cold phosphate-buffered saline (PBS) and processed for histological analysis.

### Histology

Mouse colon tissues were fixed in 10% buffered formalin for 4 h as ““swiss rolls”, which were further dehydrated, embedded in paraffin, and sectioned at 5 μm thickness. Histological scoring of tumour grades was performed on Hematoxylin and eosin (H&E) stained tissue sections in a blinded fashion according to a previously described protocol (30).

### Immunohistochemistry and immunofluorescence

Immunohistochemistry and immunofluorescence of paraffin-embedded sections were performed as previously described (30). For immunohistochemistry, anti-CD206 (Cat# ab64693, Abcam) was used as a primary antibody (Ab) followed by biotin secondary and HRP-conjugated Abs. Immunohistochemical staining was performed with DAB followed by counterstaining with hematoxylin. Quantification of positive cells (CD206+) was performed with ImageJ, a public domain Java image-processing program (National Institutes of Health) as per the procedure described previously (30). For multiplex immunofluorescence of mouse colon tissues, anti-CD8 (Cat# 38553, Cell Signaling Technology) and anti-FOXP3 (Cat# 62072, Cell Signaling Technology) were used as primary Abs. For multiplex immunofluorescence of CRC TMA, anti-ILK (Cat# ab52480, Abcam) and anti-CD11b (Cat# 49420, Cell Signaling Technology) were used as primary Abs. The SignalStain Boost IHC Detection Reagent (HRP, rabbit, 8114; Cell Signaling Technology) was used as a secondary Ab, and the staining was performed with Opal 670 (PerkinElmer, Boston, MA) for Anti-CD8 and Opal 570 (PerkinElmer) for Anti-FOXP3. All slides were mounted with SlowFade Gold Antifade Mountant with DAPI (ThermoFisher Scientific). Stained serial sections of the whole colon was scanned on a Aperio CS2 (Leica). Quantification of CD8+, FOXP3+, ILK+ and CD11b+ cells was performed manually on the high-powered fields (HPFs) of the stained serial sections of the whole colon or TMA using ImageJ, as described previously (30). All images were quantified in a blinded fashion.

### Real-time quantitative PCR

Total RNA was extracted by using the RNA mini columns (GE Healthcare) according to the manufacturer’s protocol. The concentration and purity of RNA was determined based on the absorbance ratio at 260/280 (nm). 1 μg RNA was used for cDNA synthesis in a total volume of 20 ul using Superscript First Strand Synthesis System (Invitrogen) according to the manufacturer’s protocol. Quantitative real time PCR (qRT-PCR) was performed as previously described (ref). Briefly, qRT-PCR was performed in 10 ul containing 10 ul Power SYBR Green PCR master mix (Applied Biosystems), 0.5 uM of each primer (forward and reverse), and 1–2 ul of cDNA template. qRT-PCT performed in the 7900HT Fast Real-Time PCR System (Applied Biosystems) and reactions were performed at 90°C for 8 min 30 s, followed by 40 cycles of 94°C for 15 s, and 60°C for 1 min. A melting curve was generated at the end to ensure uniformity of the product and the fold changes were obtained by converting the logarithmic scale to an exponential scale (2^-ΔΔCT^). Expression levels were normalized based on the levels of the housekeeping gene 18s. The following primers were used:

ARG1forward primer, 5´-CAGAAGAATGGAAGAGTCAG-3´; ARG1reverse primer, 5´-CAGATATGCAGGGAGTCACC-3´; YM1 forward primer, 5´- GAAGCTCTCCAGAAGCAATCCT-3´; YM1 reverse primer, 5´- GCCTGTCCTTAGCCCAACTG-3´; FIZZ1 forward primer, 5´- CCTGCTGGGATGACTGCTAC-3´; FIZZ1reverse primer, 5´- CCACTCTGGATCTCCCAAGA-3´; 18s forward primer, 5´- GTAACCCGTTGAACCCCATT-3´, and 18s reverse primer, 5´- CCATCCAATCGGTAGTAGCG -3´.

### Western blot analysis

Western blot analysis was carried out as described previously (29, 30). The membrane was immunoblotted with the following primary antibodies: rabbit polyclonal antibody against Arginase-1 (Cat. No. 9819, Cell Signaling Technology, Danvers, MA), iNOS (Cat. No. 2982, Cell Signaling Technology, Danvers, MA) and pan-actin mouse monoclonal antibody (NeoMarker, Fremont, CA). Secondary antibodies used were anti-rabbit Alexa-680- and anti-mouse Alexa-760-conjugated antibodies (Invitrogen, Carlsbad, CA). An Odyssey® Infrared Imaging System (LI-COR® Biosciences, Lincoln, NE) was used to capture the fluorescence signals which were converted into grayscale images with a white background.

### Human primary tumour samples and tissue microarray

Tissue microarray slides containing primary human colon tumours and adjacent normal tissues were obtained from the Monash Biobank facility of Monash Health, Monash Medical Centre, Melbourne, Australia. All samples derived from the Monash Biobank facility were obtained with informed consent under institutional review board–approved protocols. For IHC analysis, 13 cases of tumours along with adjacent normal tissues were examined. Quantification of ILK+ and CD11b+ cells was performed manually on the high-powered fields (HPFs) of the stained sections using ImageJ, as described previously (30).

### Statistical analysis

All data are presented as mean ± SD. Student’s *t* test or paired t test was performed for inter-group comparisons. Kaplan-Meier method was used to estimate median overall survival time, and P values were calculated using log-rank test. “n” represents number of mice or cells used in each experiments, as indicated in the figure legends. Quantifications on blinded histological samples were performed by counting/measuring microscopic fields (high-powered fields, HPFs, where indicated) as indicated in the legends. Statistical analysis was performed using Prism 9.0 (GraphPad Software La Jolla, California USA).

## Data availability statement

The data presented in the study are deposited in the figshare depository (https://figshare.com), accession code is 10.6084/m9.figshare.24534073.

## Ethics statement

The studies involving humans were approved by Monash Biobank facility of Monash Health, Monash Medical Centre, Melbourne, Australia. The studies were conducted in accordance with the local legislation and institutional requirements. The human samples used in this study were acquired from gifted from another research group. Written informed consent for participation was not required from the participants or the participants’ legal guardians/next of kin in accordance with the national legislation and institutional requirements. The animal study was approved by Monash University Animal Ethics Committee, Monash University, Australia. The study was conducted in accordance with the local legislation and institutional requirements.

## Author contributions

AA: Conceptualization, Data curation, Formal Analysis, Investigation, Methodology, Project administration, Writing – original draft. SA: Data curation, Formal Analysis, Writing – review & editing. RF: Resources, Writing – review & editing. BW: Validation, Writing – review & editing.

## Supplementary material

Supplementary Figure 1 ILK is required for partial M1 macrophage polarization. q-RT-PCR analyses of M1-specific genes encoding TNF-α, iNOS and CCL2 in both peritoneal (A, B, C) and bone-marrow derived macrophages (BMDM) (D, E, F) isolated from WT and myeloid-ILK deficient (M-ILK KO) mice are shown. Analyses were carried out under both resting and M1-polarised conditions (refer to Materials and Method section). Results are representative of three independent experiments and are shown as mean ± SD. *n* = 5 per genotype. **, *p* < 0.01; ***, *p* < 0.001; Student’s *t* test.

Supplementary Figure 2 ILK is not required for iNOS expression during M1 macrophage polarization. Western blot analysis showing iNOS protein levels in WT and ILK-KO bone-marrow derived macrophages (BMDMs) undergone M1 polarization as described in Materials and Methods. *n* = 3 per genotype.

Supplementary Figure 3 ILK is required for Arginase-1 expression during M2 macrophage polarization. Western blot analysis showing Arginase-1 protein levels in WT and ILK-KO bone-marrow derived macrophages (BMDMs) undergone M2 polarization as described in Materials and Methods. *n* = 3 per genotype.
